# Combined Immunotherapy in Treating Patients with Advanced Hepatocellular Carcinoma

**DOI:** 10.3390/biomedicines13122849

**Published:** 2025-11-21

**Authors:** Karen Hoi Lam Li, Roland Leung, Bryan Cho Wing Li, Tan To Cheung, Thomas Yau

**Affiliations:** 1 Department of Medicine, Queen Mary Hospital, The University of Hong Kong, Hong Kong, China; 2 Centre of Cancer Medicine, School of Clinical Medicine, The University of Hong Kong, Hong Kong, China; 3 Department of Surgery, Queen Mary Hospital, The University of Hong Kong, Hong Kong, China

**Keywords:** immunotherapy, immune checkpoint inhibitors, advanced hepatocellular carcinoma, HCC

## Abstract

Advanced hepatocellular carcinoma (HCC) exhibits a poor prognosis. Immunotherapy has emerged as a major player for both the upfront treatment of advanced HCC and disease progression on prior systemic therapies. In the first-line treatment of advanced HCC, immunotherapy demonstrated superior efficacy outcomes compared to tyrosine kinase inhibitors and a favourable safety profile. Initial treatment strategies of single-agent immune checkpoint inhibitors (ICIs) yielded only limited clinical activity. A deeper understanding of the hepatic tumour microenvironment and immunotolerance has driven the development of biologically relevant immunotherapy combinations. These combinations, which include antiangiogenic agents or dual ICIs targeting both PD-1/PD-L1 and CTLA-4, are the focus of current research. Recently published clinical trials involving ICI-based combination therapies achieved improved treatment outcomes, continuing to reshape the treatment paradigm for advanced HCC. While different immunotherapy combinations have shown variable efficacy in augmenting anti-tumour immunity, they inevitably increase toxicity and costs. Furthermore, the search for predictive biomarkers remains an unmet challenge in advanced HCC. In this review, we will summarise the notable advances in immunotherapy for the treatment of advanced HCC, discuss the underlying immune microenvironment and rationale for combinations, and explore opportunities for novel therapeutic targets beyond conventional immune checkpoints to overcome immunotherapy resistance.

## 1. Introduction

**Figure 1:**
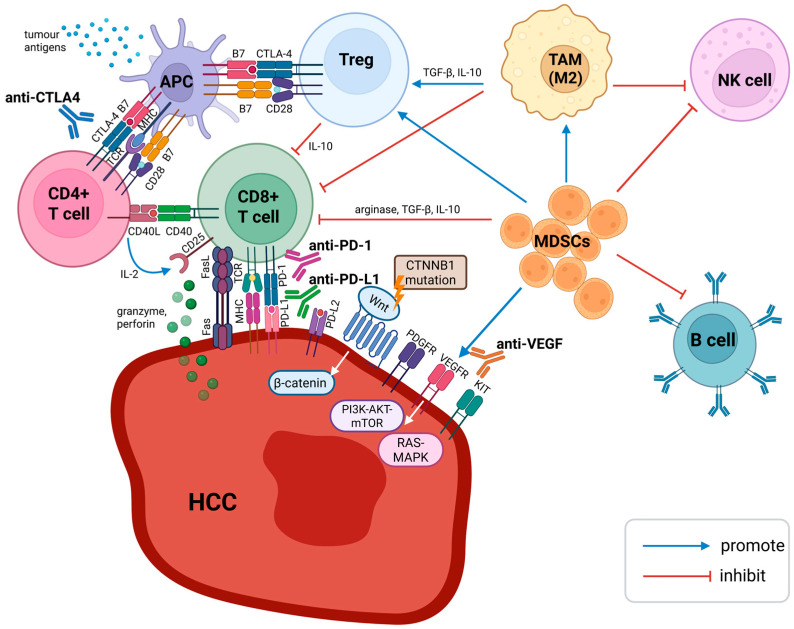
Immune microenvironment of HCC. APCs serve as an important link between the innate and adaptive immune responses. Dendritic cells are specialised APCs, which take up tumour antigens as peptide fragments. Antigen is displayed on MHC and recognised by TCR on CD4+ T cells. Interaction between B7 protein (CD80/86) on dendritic cells and CD28 on CD4+ T cells produces a costimulatory signal, whereas binding B7 protein with CTLA-4 results in an inhibitory response. CTLA-4 is also constitutively expressed on Tregs and helps maintain immune tolerance. A CTLA-4-blocking antibody would enhance CD4+ T cell activation and suppress the activity of Tregs. CD8+ T cells recognise tumour antigens presented by MHC. CD8+ T cells mediate cytotoxicity through exocytosis and the Fas/FasL pathway. Activated CD8+ T cells display PD-1 on their surface, which interacts with PD-L1 and PD-L2 on tumour cells to generate coinhibitory signals. PD-1- and PD-L1-blocking antibodies enhance the activity of CD8+ T cells in peripheral tissues and inside HCC. VEGF activation promotes angiogenesis, leading to HCC progression and metastasis. CTNNB1-mutated HCC with Wnt/β-catenin pathway activation has been reported to be resistant to immunotherapy. Dysregulated Wnt/β-catenin signalling is a major contributor to MASLD-HCC pathogenesis. Aberrant activation of PI3K-AKT-mTOR and RAS-MAPK pathways is implicated in HBV-related HCC. Treg, MDSC, and TAM (the M2 subtype) are responsible for the immunosuppressive tumour microenvironment in HCC. Abbreviations: antigen-presenting cell (APC), anti-vascular endothelial growth factor (anti-VEGF), cytotoxic T lymphocytes (CD8+ T cells), cytotoxic T-lymphocyte-associated protein 4 (CTLA-4), helper T cells (CD4+), myeloid-derived suppressor cells (MDSC), natural killer cells (NK cells), programmed cell death ligands 1 (PD-L1), programmed cell death protein-1 (PD-1), regulatory T cell (Treg), tumour-associated macrophage (TAM). Created 2025. https://BioRender.com/27hw1dt (accessed on 18 November 2025).

Primary liver cancer is a significant global health burden, ranking as the third leading cause of cancer death worldwide [1]. Hepatocellular carcinoma (HCC) accounts for 75–85% of primary liver cancer [1]. HCC classically develops in the background of chronic inflammation [2]. Hepatitis B virus (HBV) infection accounts for the majority of HCC cases in Asia and Africa, whereas chronic hepatitis C virus (HCV) infection is the predominant aetiology in patients with HCC across North America, Europe and Japan. Alcohol-related liver disease and metabolic dysfunction-associated steatotic liver disease (MASLD) are the other notable risk factors for HCC [3,4]. Surveillance for HCC in MASLD is less effective and less widely implemented. Compared to viral hepatitis-related HCC, MASLD-related HCC often develops in the absence of liver cirrhosis, and presents with larger tumour nodules at diagnosis and often with intrahepatic or distant metastases [5]. Given the liver’s constant exposure to antigens from the gastrointestinal tract, immunotolerance is crucial and is achieved via innate and adaptive immune responses [6,7]. The immune microenvironment plays a critical role in the pathogenesis of HCC [2]. A shift towards tumour immunotolerance is associated with the development and progression of HCC [8]. Evading immune destruction is one of the hallmarks of cancer [9]. The cancer-immunity cycle was conceptualised in 2013. It showed that T cells do not operate independently, but rather, they act in a sequential series of events, some of which extend beyond the immune system and tumour microenvironment [10,11]. Contemporary understanding of the cancer-immunity cycle acknowledges the essential role of the tumour microenvironment, especially dendritic cells, which play a key role in modulating and maintaining the anti-tumour T cell response (Figure 1).

Immunotherapies aim to overcome the immunosuppressive environment dominated by regulatory T cells (Tregs), myeloid-derived suppressor cells (MDSCs), and tumour-associated macrophages (TAMs) [2]. Programmed cell death protein-1 (PD-1) is a cell-surface protein first discovered in 1992. PD-1 is typically expressed on T-cells and functions to downregulate T cell responses, thereby preventing autoimmune disease. Programmed cell death ligands 1 and 2 (PD-L1 and PD-L2) are the ligands for PD-1. PD-L1 is expressed on antigen-presenting cells, immune cells and tumour cells. PD-1 and PD-L1 interaction results in intratumoral T-cell exhaustion and eventually immune invasion. Cytotoxic T-lymphocyte-associated protein 4 (CTLA-4) is another inhibitory receptor expressed exclusively on T cells and was first discovered in 1987 [12,13,14]. CD80 (B7-1) and CD86 (B7-2) are ligands for CTLA-4, which when bound to CTLA-4, leads to T cell inhibition. The discovery of PD-1 and CTLA-4 immune checkpoints was a significant breakthrough, ultimately leading to James P. Allison and Tasuku Honjo receiving the 2018 Nobel Prize in Physiology or Medicine [15]. While PD-1 and PD-L1 blocking antibodies enhance the activity of cytotoxic T lymphocytes (CD8+ T cells) in peripheral tissues and inside tumours, CTLA-4-blocking antibodies activate CD8+ T cells and suppress the activity of Tregs in the initial T cell activation stage [16,17]. Anti-PD-1 antibodies inhibit PD-1 interaction with both PD-L1 and PD-L2, while anti-PD-L1 antibodies prevent PD-1 from binding to PD-L1 but allow interaction with PD-L2. Immunotherapies are associated with a distinct spectrum of adverse events known as immune-related adverse events (irAE), and the pathophysiology is postulated to be linked to the function of immune checkpoints in preserving immunological homeostasis [18]. Key mechanisms of irAE include T cell activation, increasing levels of preexisting autoantibodies, cytokine involvement, and cross-reactivity [18]. In general, CTLA-4-blocking agents are associated with more high-grade immune-related toxicities than PD-1/PD-L1 blockade [19,20].

Macrovascular invasion (MVI) in HCC most often presents as portal vein tumour thrombosis (PVTT). HCC with PVTT is usually associated with compromised liver function, portal hypertension and higher rates of metastatic spread. MVI predicts poorer outcomes, particularly those involving the lobar branches or main trunk of the portal vein (Vp3 or Vp4). Patients with PVTT have a guarded prognosis, with variable OS around 5 to 15 months depending on treatment modality [21,22,23]. Most clinical trials of immunotherapy in advanced HCC exclude patients with PVTT [24,25]. A systematic review and meta-analysis show that ICI-treated HCC patients with MVI have inferior PFS and OS, while the incidence of irAE is not significantly increased [26].

**Figure 2:**
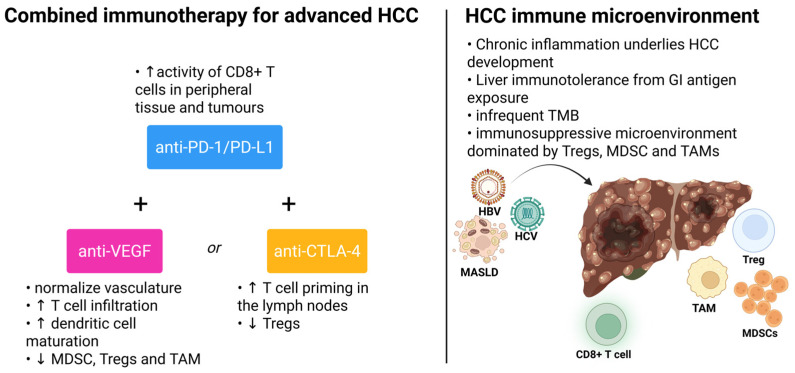
Combined immunotherapy for advanced HCC and tumour immune microenvironment. Created 2025. https://BioRender.com/1iubbzk (accessed on 18 November 2025).

Clinical trials commonly use Barcelona Clinic Liver Cancer (BCLC) staging to define their target populations. Advanced HCC refers to BCLC-C or BCLC-B, where the tumour has progressed after locoregional therapies or are too extensive to be treated selectively by locoregional therapies [27]. In these trials, most patients are Child–Pugh A with good performance statuses. Furthermore, those with prior or active autoimmune diseases or a history of solid organ transplant are excluded from trials involving immunotherapies (Figure 2).

## 2. The First Breakthrough: Antiangiogenic Agents

Sustained angiogenesis is one of the hallmarks of cancer first described in 2000 [28]. Neovascularisation has also been implicated in the pathogenesis of HCC [29]. In HCC and other solid tumours, angiogenesis relies on the activation, proliferation and migration of endothelial cells. Consequently, initial efforts have been focused on the therapeutic inhibition of angiogenesis in HCC. Angiogenesis inhibitors in HCC can generally be divided into antibody-based treatments and tyrosine kinase inhibitors (TKIs). Sorafenib, an oral TKI targeting RAF, VEGFR, PDGFRβ and RET, emerged as the first targeted therapy for advanced HCC based on the landmark SHARP study in 2008 [16]. In this study, median overall survival (OS) was 10.7 months in the sorafenib group, compared to 7.9 months in the placebo group (HR 0.69, *p* < 0.001) [30]. Sorafenib was the first systemic therapy approved by the Food and Drug Administration (FDA) for the treatment of patients with advanced HCC. Lenvatinib was another multitargeted oral TKI, approved in 2018 based on non-inferiority to sorafenib in overall survival in the REFLECT study [31]. Lenvatinib targets VEGFR1-3, FGFR1-4, PDGFRα, KIT and RET [16]. It should be emphasised that in the pre-immunotherapy era, achieving non-inferiority was a substantial hurdle [32,33]. TKIs confer modest survival benefits and are associated with considerable adverse events. Therefore, there is an unmet need for better advanced HCC treatment options.

## 3. The Game Changer: Evolving Immunotherapy Landscape

A more comprehensive understanding of the pathogenesis, tumour microenvironment and immune system made immune checkpoints attractive therapeutic targets for advanced HCC. Several positive randomised controlled trials have recently accelerated a paradigm shift towards immunotherapy-based treatments for advanced HCC. Immunotherapies for the treatment of advanced HCC generally involve an anti-PD-1/PD-L1 agent as monotherapy or in combination with an antiangiogenic or anti-CTLA-4 agent [3]. Commonly used immunotherapies for HCC include anti-PD-1 (nivolumab, pembrolizumab, camrelizumab, toripalimab and sintilimab), anti-PD-L1 (atezolizumab, durvalumab and tislelizumab) and anti-CTLA-4 (ipilimumab and tremelimumab) antibodies. The main efficacy outcomes of selected immunotherapy trials in advanced HCC are summarised in Table 1, which will be discussed.

### 3.1. Immune Checkpoint Inhibitor Monotherapy

In the phase II KEYNOTE-224 trial, second-line pembrolizumab achieved an overall response rate (ORR) of 17% in patients with advanced HCC and gained accelerated approval in 2018 [34,35]. In the confirmatory Phase III Keynote-240 trial, pembrolizumab as second-line therapy did not achieve statistical significance in progression-free survival (PFS) and OS improvement compared with placebo, although a favourable risk–benefit ratio was demonstrated [36]. In contrast, in Asian patients with previously treated advanced HCC, pembrolizumab was associated with statistically significant and clinically meaningful improvement in OS, PFS, and ORR compared with placebo in KEYNOTE-394 [37]. The design of KEYNOTE-394 and KEYNOTE-240 was identical, except that KEYNOTE-394 comprised an entirely Asian population, while KEYNOTE-240 was a global study. Notably, the majority of subjects in KEYNOTE-240 were non-Asians with a non-viral aetiology of HCC [38,39]. Despite the negative readout of KEYNOTE-240, FDA’s Oncologic Drugs Advisory Committee (ODAC) voted to keep the approval for pembrolizumab [40]. CheckMate-040 is a Phase I/II study involving six cohorts, investigating nivolumab as a monotherapy or in combination with other agents for patients with advanced HCC [41]. Nivolumab monotherapy demonstrated clinical benefit in patients with advanced HCC in CheckMate-040 and led to nivolumab being investigated in the first-line treatment in CheckMate-459 against sorafenib, which was the standard of care at that time [42]. CheckMate-459 was the first phase 3 trial to evaluate a single-agent PD-1 inhibitor in the first-line treatment setting. Despite the fact that frontline nivolumab did not achieve a significant improvement in OS when compared with sorafenib (mOS 16.4 months for nivolumab vs. 14.7 months for sorafenib, HR 0.85, *p* = 0.075), it showed a favourable safety profile and represented a potential treatment option for patients contraindicated for antiangiogenic agents [42]. It is noteworthy that nivolumab demonstrated an ORR of 12% and a disease control rate of 55% in patients with Child–Pugh B cirrhosis (B7-B8) in the Phase I/II CheckMate 040 Cohort 5 [43]. In RATIONALE-301, tislelizumab demonstrated non-inferiority to sorafenib in terms of OS [44]. Nevertheless, the limited efficacy of single-agent immunotherapy for HCC warranted the need for more effective treatment strategies.

### 3.2. Combining Immune Checkpoint and Angiogenesis Blockade

Antiangiogenic agents are broadly classified into anti-vascular endothelial growth factor (anti-VEGF) antibodies and multitargeted TKIs. IMbrave150 was the first phase III randomised trial to show a significant improvement in overall survival with the combination of atezolizumab and bevacizumab compared with sorafenib and was FDA-approved in 2020 [45]. In the updated analysis, atezolizumab plus bevacizumab yielded a median OS of 19.2 months, with a 5.8 month improvement over the 13.4 months achieved by sorafenib (HR 0.66, *p* < 0.001), and a nearly three-fold higher overall response rate (30% vs. 11%) [46] Bevacizumab is a humanised monoclonal antibody against circulating VEGF-A that normalises the vasculature, increases T cell infiltration and promotes dendritic cell maturation [29,47]. Phase II studies have indicated that bevacizumab, as a monotherapy, exhibits only minimal activity against advanced HCC, like most other solid tumours [48]. It has been demonstrated that multiple cell types within the tumour microenvironment with established immunosuppressive functions contribute to angiogenesis through the production of various growth factors [49]. Angiogenesis inhibitors also downregulate the activity of MDSC, Tregs and TAM, shifting the tumour microenvironment from immunosuppressive to immune-permissive [50]. Combining antiangiogenic agents and immunotherapy is thus biologically relevant. Notably, the incidence of upper gastrointestinal bleeding was 7% in the atezolizumab plus bevacizumab group, as compared with 4.5% in the sorafenib group [45]. In view of the increased bleeding risk associated with bevacizumab, an upper endoscopy within 6 months of initiation of therapy was mandated for patients considered for atezolizumab and bevacizumab. Baveno VI and VII criteria have yet to be validated for HCC patients [51,52]. IMbrave150 also excluded patients on anticoagulants or with high bleeding risk [45]. Exploratory analysis of IMBrave 150 shows the consistent benefit of atezolizumab plus bevacizumab over sorafenib in the Vp4 subgroup (mOS 7.6 vs. 5.5 months, HR 0.62, *p* = 0.104) [53]. A further prospective study involving Korean patients found that high levels of antidrug antibodies against atezolizumab at 3 weeks may be associated with inferior clinical outcomes when treated with atezolizumab and bevacizumab [54]. ORIENT-32 is a phase II/III study that demonstrated that sintilimab plus IBI305 (a bevacizumab biosimilar) was superior to sorafenib in OS, PFS and ORR in all patients in China. Patients with Child–Pugh class B7 cirrhosis were allowed in ORIENT-32 [55].

In CARES-310, camrelizumab plus rivoceranib improved OS, PFS and ORR compared to sorafenib in a predominantly Asian population, despite a higher incidence of grade 3–4 TRAE in the camrelizumab plus rivoceranib arm than in the sorafenib arm (81% vs. 52%) [56,57]. Rivoceranib is a VEGFR2-targeted TKI. VEGFR2 is expressed on endothelial cells and activated upon binding to VEGF-A [16]. In the phase III LEAP-002 global study, pembrolizumab plus lenvatinib for patients with treatment-naïve advanced hepatocellular carcinoma had improved ORR compared with lenvatinib monotherapy but failed to improve the dual primary endpoints OS or PFS. Long-term follow-up of LEAP-002 showed that the median OS was 21.1 months with pembrolizumab plus lenvatinib and 19.0 months with lenvatinib, HR 0.80. The 5 year OS rate was almost doubled in patients receiving the combo compared with lenvatinib, 19.7% vs. 10.7% [58,59]. LEAP-002 mandated an upper endoscopy within 3 months of randomisation, and patients with main portal vein invasion were excluded. While the majority of patients in LEAP-002 were non-Asian, with 63% in the experimental arm having a viral aetiology, the prespecified subgroup analysis of overall survival showed signals favouring the combination of pembrolizumab and lenvatinib in patients with HBV aetiology [58]. In COSMIC-312, cabozantinib (a multikinase inhibitor of RTKs including AXL, FIT-3, KIT, MET, RET, VEGFR 1-3) plus atezolizumab improved PFS and ORR over sorafenib but showed no difference in OS. As both PFS and OS were dual primary endpoints, COSMIC-312 can be regarded as a “semi-positive” trial. The mPFS was 6.9 months for the combination of cabozantinib plus atezolizumab vs. 4.3 months for sorafenib (HR 0.74), ORR 13% vs. 5%, OS 16.5 vs. 15.5 months (HR 0.98, *p* = 0.87) [60,61]. Patients in the cabozantinib plus atezolizumab arm experienced higher dose reductions than those in the sorafenib arm (62 vs. 43%). There was also a notable disparity in post-progression treatment patterns, with an unexpectedly lower proportion of patients in the cabozantinib plus atezolizumab arm (26%) receiving subsequent therapies compared to the sorafenib arm (42%). The underlying reason, including differential liver function at the cessation of cabozantinib plus atezolizumab versus sorafenib, remains unclear.

It is also noteworthy that the proportion of patients with HBV aetiology was lower in COSMIC-312 than in IMbrave150, LEAP-002 and CARES-310. As with LEAP-002, subgroup analysis of COSMIC-312 suggested a potential benefit with atezolizumab and cabozantinib in patients with HBV aetiology. A meta-analysis of IMbrave150, CheckMate-459, and KEYNOTE-240 showed that HBV- and HCV-related HCC derive greater benefit than non-viral HCC from immunotherapy [62]. Recently published HEPATORCH shows that toripalimab plus bevacizumab significantly improves PFS and OS as compared with sorafenib [63]. This combination has been approved in China. The combination of an immune checkpoint inhibitor with an anti-VEGF antibody is a promising therapeutic strategy to potentiate anti-tumour immunity for patients with advanced HCC, and further translational studies are needed to characterise patients who benefit most from this combination. Despite the proven efficacy of oral multitargeted TKIs sorafenib and lenvatinib in the first-line treatment of advanced HCC, their combination with immune checkpoint inhibitors has yielded mixed results. CARES-310 is the first positive international phase III study of the combination with an immune checkpoint inhibitor and an oral small molecule tyrosine kinase inhibitor. However, rivoceranib exhibits high selectivity for VEGFR2, and is, therefore, distinguished from other multitargeted TKIs used for the treatment of HCC.

### 3.3. Combining CTLA-4 and PD-1 Blockade

The combination of PD-1/PD-L1 and CTLA-4 targeting antibodies has shown distinct and complementary effects. CTLA-4 primarily regulates T-cell activation at the priming stage in tumour-draining lymph nodes involving CD28-mediated T-cell co-stimulation, while PD-1 inhibition primarily occurs at the tumour site during the effector phase. CTLA-4 blockade restores the positive CD28 costimulatory signals through B7 binding and down-regulates immunosuppressive Tregs. On the other hand, PD-1 directly phosphorylates CD28 via SPH2 [64,65]. This shared targeting of CD28 represents a functional convergence in their regulatory roles.

The STRIDE regimen, consisting of a single dose of tremelimumab with durvalumab every four weeks in patients with unresectable HCC, was evaluated in the phase III HIMALAYA study. Patients with main portal vein thrombosis and gastrointestinal bleeding within the last 12 months were excluded. An earlier Phase I/II study showed that a single priming dose of tremelimumab demonstrated a saturable correlation between tremelimumab exposure and CD8+ T cell response, suggesting that further repeated tremelimumab exposure does not necessarily result in further CD8+ T cell proliferation [66]. The primary endpoint, OS for STRIDE regimen versus sorafenib, was met. The mOS was 16.43 with STRIDE, as compared with 13.77 months with sorafenib (HR 0.78, *p* = 0.0035). STRIDE also increased the ORR compared to sorafenib (20.1% vs. 5.1%). However, mPFS was similar between STRIDE and sorafenib. This is opposite to COSMIC-312, where PFS was not translated to OS. Updated analysis from HIMALAYA showed a sustained OS benefit of STRIDE versus sorafenib, with a 5 year OS rate of 19.6% versus 9.4%. Among patients who received STRIDE, the OS benefit was more pronounced in patients who experienced disease control and any degree of tumour shrinkage [67]. FDA approved tremelimumab in combination with durvalumab for unresectable HCC in 2022 [68]. In the HIMALAYA study, durvalumab monotherapy demonstrated non-inferiority to sorafenib (HR 0.86, 95.67% CI 0.73–1.03; non-inferiority margin, 1.08) for patients with unresectable HCC [69]. The ongoing SIERRA phase IIIb trial will help to address STRIDE regimens in broader populations with advanced HCC, including those with Child–Pugh B cirrhosis (B7-B8), performance status 2 or Vp4 [70].

CheckMate-040 trial cohort 4 is a phase I/II study that randomised patients with advanced HCC to receive the combination of nivolumab 1 mg/kg plus ipilimumab 3 mg/kg every 3 weeks for 4 doses, then nivolumab 240 mg every 2 weeks (Arm A), nivolumab 3 mg/kg plus ipilimumab 1 mg/kg every 3 weeks for 4 doses, then nivolumab 240 mg every 2 weeks (Arm B); or nivolumab 3 mg/kg every 2 weeks, plus ipilimumab 1 mg/kg every 6 weeks (Arm C). Patients in arm A achieved the longest median OS at 22.8 months, compared to 12.5 months in arm B; and 12.9 months in arm C. ORR was 32% in arm A, 27% in arm B and 29% in arm C. The long-term survival benefit was maintained with a 5 year OS rate of 29% in arm A. Notably, grade 3–4 TRAE was higher in arm A than arm B, 53% vs. 29%, respectively [71,72,73]. Higher doses of ipilimumab monotherapy in advanced melanoma and in combination with nivolumab in small cell lung cancer have also been reported to be correlated with improved survival outcomes and higher rates of irAE [74,75]. The encouraging result of nivolumab plus ipilimumab in CheckMate-040 supported further investigation of this regimen as a first-line treatment option in the CheckMate-9DW trial. In this study, patients with unresectable systemic therapy-naïve HCC were randomised to receive nivolumab 1 mg/kg plus ipilimumab 3 mg/kg for four cycles, followed by nivolumab 480 mg every 4 weeks, or the investigator’s choice of either lenvatinib or sorafenib. The median OS for patients treated with nivolumab plus ipilimumab was 23.7 months, as compared to 20.6 months in the lenvatinib or sorafenib arm (HR 0.79, *p* = 0.018), which is the longest reported OS to date in this setting. The OS benefit was consistent across clinically relevant subgroups, regardless of aetiology and PD-L1 CPS status. A post hoc analysis showed that nivolumab plus ipilimumab conferred overall survival benefits versus lenvatinib (HR 0.77) or sorafenib (0.42) individually after propensity score matching. Although PFS was comparable between the two groups, the nivolumab plus ipilimumab group had numerically higher PFS rates at 18 and 24 months, as well as a more prolonged PFS on next-line therapy of 19.3 months versus 15.4 months in the lenvatinib or sorafenib group, although 35% of patients received subsequent immunotherapy. The combination of nivolumab and ipilimumab compared to lenvatinib and sorafenib resulted in a superior objective response rate (36% vs. 13%, *p* < 0.0001), and longer duration of response (30.4 vs. 12.9 months). For patients who received nivolumab and ipilimumab, achieving complete response or partial response as the best overall response at 24 months is predictive of excellent survival benefits, without reaching the median OS (NR) (95% CI 44.4 months-NR) [76]. Grade 3–4 TRAEs occurred at similar rates between ipilimumab plus nivolumab and lenvatinib or sorafenib group (41% vs. 42%) [77].

Both the STRIDE regimen and nivolumab plus ipilimumab demonstrated a favourable risk–benefit profile for patients with advanced HCC, regardless of albumin–bilirubin (ALBI) grade [78,79,80]. Patients with Vp4 were excluded from both the HIMALAYA and CheckMate-9DW studies. Based on the results of CheckMate-040 cohort 4, the FDA granted accelerated approval for nivolumab and ipilimumab for HCC patients previously treated with sorafenib, with nivolumab 1 mg/kg plus ipilimumab 3 mg/kg every 3 weeks (four doses), then nivolumab monotherapy 240 mg every 2 weeks or 480 mg every 4 weeks in 2020 [81]. The nivolumab and ipilimumab combination was also approved for first-line treatment of unresectable or metastatic HCC, according to CheckMate-9DW in 2025 [82]. Overall, the robustness of the combined CTLA-4 and PD-1/PD-L1 blockade in front-line treatment of advanced HCC was nicely demonstrated in both HIMALAYA, with durable 5 year OS data, and as well as in CheckMate-9DW with a deep depth of response. In 2024, the FDA approved nivolumab and hyaluronidase-nvhy for subcutaneous injection across all approved solid tumours, including HCC, in adults. However, nivolumab and hyaluronidase-nvhy is not indicated for concurrent use with ipilimumab. Nivolumab and hyaluronidase-nvhy may be used for advanced HCC in place of intravenous nivolumab monotherapy [83]. Hence, in summary, subcutaneous administration of immunotherapy has the potential benefits of reducing infusion chair occupancy, reducing injection site complications and shortening administration time.

### 3.4. The Search for Biomarkers

The lack of reliable predictive biomarkers to guide immunotherapy in HCC remains a challenge [84]. Although HCC in cirrhotic patients can be diagnosed based on non-invasive imaging, such as multiphasic liver protocol CT, tumour biopsy is recommended for patients enrolling in clinical trials to facilitate biomarker development [3].

Current evidence suggests that immunotherapies may confer better outcomes for HCC related to viral hepatitis than non-viral aetiologies. This may be explained by the fact that CD8+ T cells in MASLD-related HCC fail to mount immune surveillance, paradoxically leading to tissue damage, and promoting a protumorigenic environment [85]. The Wnt/TGF-β proliferation subclass in MASLD-related HCC has been associated with resistance to immunotherapy [86]. On the other hand, the abundance of viral antigens and a distinct microenvironment may lead to improved outcomes of viral HCC patients treated with immunotherapy [2,62,87]. HBV-related HCC is characterised by the upregulation of PI3K-AKT-mTOR, RAS-MAPK, MET and IGF pathways, as well as frequent TP53 mutations [88]. Although the influence of hepatocellular carcinoma aetiology on immune checkpoint inhibitor response and survival has been documented in preclinical studies and post hoc analysis of randomised controlled trials, viral aetiology does not always predict treatment outcomes, and stratification based on disease viral aetiology has not been validated [89,90]. We should also be mindful of the heterogeneity of the definition of viral aetiologies across trials, for instance, the variability of HBV and HCV serology tests used. The global burden of viral hepatitis-related HCC is decreasing due to surveillance programmes and the availability of effective antivirals. On the other hand, the prevalence of MASLD-related HCC is rising. Patients with HCC related to MASLD often have multiple cardiovascular risk factors, which may affect HCC treatment; for example, this could necessitate a more cautious use of antiangiogenic agents. Future clinical trials should consider prespecified subgroup analyses of underlying aetiologies of HCC, particularly viral hepatitis and MASLD, to better address whether HCC treatment should be aetiology-based [5,91].

Advancements in multiomic profiling enable molecular reclassification of HCC, which subsequently facilitates the development of subclass-specific therapeutic agents [92]. Based on transcriptomic analysis, HCC can be classified into proliferation and non-proliferation classes. The proliferation class is characterised by tumours displaying aggressive traits, such as poor histological differentiation, vascular invasion and elevated α-fetoprotein (AFP). On the other hand, non-proliferation class HCC are less aggressive, and generally well-to-moderately differentiated with low AFP levels [88]. Immunogenomic classification divides HCC into immune-active, immune-exhausted, immune-intermediate, and immune-excluded subclasses. The abundance of helper T (CD4+) and CD8+ T cell infiltrates in immune-active HCC predicts response to immune checkpoint inhibitors [88,93]. CTNNB1-mutated HCC with Wnt/β-catenin pathway activation has been reported to be resistant to immunotherapy [94]. CTNNB1 mutations in HCC are associated with an immune-exhausted phenotype [88,93]. However, translation of molecular classifications of HCC from bench to bedside has been lagging in comparison with many other solid organ tumours. One of the reasons is that most targets identified in HCC, e.g., TERT promoter-activating mutation, TP53 loss of function, CTNNB1-activating mutation and RAS–PI3K–mTOR pathway alterations are not yet druggable [95,96]. Intra- and inter-tumour heterogeneity is another obstacle for molecular classification of HCC [97]. The predictive value of PD-L1 expression in advanced HCC is still under investigation.

Currently, all FDA-approved immunotherapies for HCC have been granted irrespective of PD-L1 expression. A meta-analysis including immunotherapy monotherapy trials shows that positive PD-L1 expression may be associated with higher response rates in advanced HCC [98]. CD8+ T cell infiltration does not reliably predict immunotherapeutic response in HCC [95]. Moreover, tumour mutation burden-high (TMB-H) is an infrequent finding in HCC, and clinical application of TMB as a biomarker in HCC is limited [84]. An exploratory analysis of GO30140 phase 1b and IMbrave150 showed that high expression of VEGF Receptor 2 and intratumoral CD8+ T cell density were associated with improved clinical outcomes, while no correlation was found between TMB and survival benefits [99]. A recent study showed that high FGF21 predicts worse survival outcomes in HCC treated with atezolizumab and bevacizumab [100]. Together, comprehensive molecular profiling, proteomic classification, and immunogenomic characterisation may be the future direction of HCC in the era of immunotherapy [95].

## 4. The Future Prospect of Immunotherapy in HCC

### 4.1. Moving Immunotherapy to Intermediate-Stage HCC

The promising results of immunotherapy in advanced HCC have sparked interest in expanding immunotherapies for intermediate-stage disease. Theoretically, moving immunotherapies to intermediate-stage disease has the advantage of incorporating effective treatment while liver function is still preserved, thereby maximising response rates and survival. The ABC-HCC trial is an ongoing phase III randomised to compare atezolizumab and bevacizumab versus transarterial chemoembolisastion (TACE) in intermediate HCC [101].

The goal of combining liver-directed therapies and immunotherapy in intermediate-stage HCC was to induce immunogenic cell death and activate dendritic cells by the release of tumoral neoantigens, thereby transforming the immunosuppressive microenvironment to an immunosupportive one [102]. Transarterial radioembolisation (TARE), followed by nivolumab in advanced HCC has demonstrated an ORR of 30% in a single-centre phase II trial [103]. Adding durvalumab and bevacizumab to TACE improved PFS compared with TACE alone for patients with unresectable HCC amenable to TACE, according to the phase III EMERALD 1 study [104]. The phase III LEAP-012 study also demonstrated PFS benefit with TACE combined with pembrolizumab plus lenvatinib in unresectable, non-metastatic HCC [105]. However, in view of the lack of mature overall survival data and higher risk of toxicity, the combination of immunotherapy with locoregional therapies should only be offered to highly selected patients with shared decision-making.

### 4.2. Novel Combinations and Targets

Advanced HCC has a high unmet clinical need due to a modest OS of under 2 years. Current research aims to improve treatment efficacy by exploring various combinatorial strategies and identifying novel therapeutic targets. In the phase I/II CheckMate-040 cohort 6, triplet therapy with nivolumab, cabozantinib, and ipilimumab yielded a mOS of 22.1 months, mPFS of 4.3 months, and ORR of 29% in the first-line treatment of advanced HCC [106]. TRIPLET is a phase II/III trial assessing the benefit of adding ipilimumab to the combination of atezolizumab and bevacizumab in patients with advanced HCC treated in the first line [107].

Novel checkpoint targets, such as TIGIT, TIM3 and LAG-3, may define the next wave of immunotherapy development for advanced HCC with the aim to enhance T-cell function and infiltration. T cell immunoreceptor with Ig and ITIM domains (TIGIT) is another coinhibitory receptor expressed on activated and exhausted T cells and natural killer (NK) cells [108]. Patients with HCC have high TIGIT expression in both CD4+ T cells and Tregs. TIGIT plays a critical role in the TIGIT–CD226–PVR axis. TIGIT, CD226, CD96 and PVRIG are expressed on T and NK cells, whereas CD155 (PVR) is often overexpressed on tumour cells. The binding of CD226 to its ligand CD155 promotes an antitumour immunity response, while TIGIT competitively interacts with CD155 to induce tumour-immune invasion. Anti-TIGIT antibodies restore the CD226–CD155 interaction. Innovative combination strategies with anti-TIGIT monoclonal antibody aim to synergise with anti-PD-1/PD-L1 to enhance anti-tumour immunity in a non-redundant manner [109,110,111]. Tiragolumab is an anti-TIGIT monoclonal antibody [112]. MORPHEUS-Liver is a phase Ib-2 study evaluating the addition of tiragolumab to atezolizumab plus bevacizumab as a first-line treatment for advanced HCC. The triplet combination yielded a significantly improved ORR (43% vs. 11%), PFS (12.3 vs. 4.2 months, HR 0.51) and OS (28.9 vs. 15.1 months, HR 0.39). Moreover, the addition of tiragolumab did not substantially increase the incidence of treatment-related or immune-mediated adverse events [112]. The results of the ongoing phase III IMbrave152/SKYSCRAPER-14 trial are eagerly awaited [110]. Lymphocyte-activation gene 3 (LAG-3) is structurally similar to CD4, found on the surface of helper T cells. With the MHC class II as its major ligand, LAG-3 plays a functional role in Treg-mediated immunosuppression [108]. RELATIVITY-106 is under way to evaluate the triple therapy nivolumab, relatlimab and bevacizumab in treatment-naïve HCC. T cell immunoglobulin domain and mucin domain 3 (TIM-3) found on CD4+ T cells negatively regulates CD4+ T cells through its ligand Galectin-9 [113]. TIM-3 is found to be upregulated in patients who develop resistance to PD-1 blockade [16,114]. Cobolimab (anti-TIM3 monoclonal antibody) plus dostarlimab has shown efficacy and safety in a phase II study in the front-line treatment of unresectable HCC [115]. Bispecific antibodies (bsAbs) are an emerging therapeutic strategy in haematological and solid organ malignancies. bsAbs are engineered to simultaneously target two different epitopes. Dual checkpoint inhibitor-blocking bsAbs aim to overcome immune checkpoint inhibitor resistance and enhance efficacy by simultaneously blocking two immune checkpoint receptors [116,117,118]. KN046 is a bsAb inhibiting both PD-L1 and CTLA-4. In a phase II trial, KN046 in combination with lenvatinib showed promising efficacy in advanced HCC, and grade ≥ 3 TRAEs were reported in 47.3% of patients [119]. ARTEMIDE-HCC01 is a phase III study evaluating rilvegostomig (anti-PD-1/TIGIT bsAb) plus bevacizumab with or without tremelimumab compared to atezolizumab plus bevacizumab [120]. The GEMINI-Hepatobiliary phase II study has a platform design and includes an HCC cohort testing of volrustomig (anti-PD-1/CTLA-4 bsAb) as a monotherapy or in combination with bevacizumab or Lenvatinib [121].

## 5. Conclusions

The introduction of immunotherapies for patients with advanced HCC has been revolutionary, leading to groundbreaking results over the past few years. A better understanding of tumour microenvironment and cancer immunity has paved the way for rational combinations of immunotherapies, which generally exhibit superior outcomes than single agents. The use of single-agent anti-PD-1/PD-L1 should be limited to patients with advanced HCC who are contraindicated to receive combination immunotherapy. Precision medicine with an emphasis on molecular profiling, biomarker-driven therapies and personalised treatment strategies is the future direction of HCC research. There are no head-to-head comparisons between anti-PD-1/PD-L1 in combination with angiogenesis inhibitors and anti-CTLA-4. Caution should be taken when comparing trials, in light of variability in trial designs, demographic factors and eligibility criteria. Given the robust evidence of immunotherapies in advanced HCC, the combination of immune therapies with locoregional therapies are being investigated in intermediate-stage HCC. Overall, this is an exciting era for immunotherapies for HCC.

## Figures and Tables

**Table 1:** Clinical trials of immunotherapy in advanced HCC.

Trial	Year of First Publication	Phase	Experimental Arm and Control Arm	PFS (Months)	OS (Months)	ORR (%)	G 3–4 TRAEs (%)
1st line							
Anti-PD-1 (Monotherapy)							
**CheckMate-459**	2022	III	Nivolumab vs. sorafenib	3.7 vs. 3.8 (HR 0.93)	16.4 vs. 14.7 (HR 0.85, *p* = 0.075)	15 vs. 7	22 vs. 49
**RATIONALE-301**	2023	III	Tislelizumab vs. sorafenib	2.1 vs. 3.4 (HR 1.11)	15.9 vs. 14.1 (HR 0.85)	14.3 vs. 5.4	22.2 vs. 53.4
**Anti-PD-1/PD-L1 and Antiangiogenic Agents (Doublet Therapy)**							
**IMbrave150**	2020	III	Atezolizumab plus bevacizumab vs. sorafenib	6.9 vs. 4.3 (HR 0.65, *p* < 0.001)	19.2 vs. 13.4 (HR 0.66, *p* < 0.001)	30 vs. 11	43 vs. 46
**ORIENT-32**	2021	II/III	Sintilimab plus a bevacizumab biosimilar (IBI305) vs. sorafenib	4.6 vs. 2.8 (HR 0.56, *p* < 0.001)	NR vs. 10.4 (HR 0.57, *p* < 0.001)	21 vs. 4	53 vs. 45
**COSMIC-312**	2022	III	Cabozantinib plus atezolizumab vs. sorafenib	6.9 vs. 4.3 (HR 0.74)	16.5 vs. 15.5 (HR 0.98, *p* = 0.87)	13.0 vs. 5	66 vs. 48
**CARES-310**	2023	III	Camrelizumab plus rivoceranib vs. sorafenib	5.6 vs. 3.7 (HR 0.52, *p* < 0.0001)	23.8 vs. 15.2 (HR 0.64, *p* < 0.0001)	25 vs. 6	81 vs. 52
**LEAP-002**	2023	III	Lenvatinib plus pembrolizumab vs. lenvatinib	8.2 vs. 8.0 (HR 0.87, *p* = 0.047)	21.2 vs. 19.0 (HR 0.84, *p* = 0.023)	26.1 vs. 17.5	62 vs. 57
**HEPATORCH**	2025	III	Toripalimab plus bevacizumab vs. sorafenib	5.8 vs. 4.0 (HR 0.69, *p* = 0.0086)	20.0 vs. 14.5 (HR 0.76, *p* = 0.039)	25 vs. 6	63 vs. 61
**Anti-PD-1/PD-L1 and Anti-CTLA-4 (Doublet Therapy)**							
**HIMALAYA**	2022	III	Tremelimumab plus durvalumab (STRIDE) vs. sorafenib	3.78 vs. 4.07 (HR 0.90)	16.43 vs. 13.77 (HR 0.76, *p* = 0.0008)	20.1 vs. 5.1	50.5 vs. 52.4
**CheckMate-9DW**	2025	III	Nivolumab plus ipilimumab vs. lenvatinib or sorafenib	9.1 vs. 9.2 (HR 0.87)	23.7 vs. 20.6 (HR 0.79, *p* = 0.018)	36 vs. 13	41 vs. 42
**Anti-PD-1/PD-L1 and Antiangiogenic Agents (Triplet Therapy)**							
**CheckMate-040 Cohort 6**	2022	I/II	Nivolumab, cabozantinib and ipilimumab	22.1	4.3	29	74
**MORPHEUS-Liver**	2025	Ib/II	Tiragolumab plus atezolizumab and bevacizumab vs. atezolizumab and bevacizumab	12.3 vs. 4.2 (HR 0.51)	28.9 vs. 15.1 (HR 0.39)	43 vs. 11	33 vs. 44
**2nd line**							
**KEYNOTE-224**	2018	II	Pembrolizumab			17	25
**KEYNOTE-240**	2019	III	Pembrolizumab vs. placebo	3.0 vs. 2.8 (HR 0.718, *p* = 0.0022)	13.9 vs. 10.6 (HR 0.781, *p* = 0.0238)	18.3 vs. 4.4	18.6 vs. 7.5
**KEYNOTE-394**	2022	III	Pembrolizumab vs. placebo	2.6 vs. 2.3 (HR 0.74, *p* = 0.032)	14.6 vs. 13.0 (HR 0.79, *p* = 0.0180)	12.7 vs. 1.3	13.3 vs. 5.9

Abbreviations: grade 3–4 treatment-related adverse event (TRAE), hazard ratio (HR).

## Data Availability

The original contributions presented in this study are included in the article. Further inquiries can be directed to the corresponding author.

## Abbreviations

The following abbreviations are used in this manuscript:

ALBI	albumin–bilirubin
APC	antigen-presenting cell
anti-VEGF	anti-vascular endothelial growth factor
BCLC	Barcelona Clinic Liver Cancer
bsAb	bispecific antibody
CPS	combined positive score
CD8+	cytotoxic T lymphocyte
CTLA-4	cytotoxic T-lymphocyte-associated protein 4
CD4+	helper T cells
HBV	hepatitis B virus
HCV	hepatitis C virus
HCC	hepatocellular carcinoma
ICI	immune checkpoint inhibitor
irAE	immune-related adverse event
LAG-3	lymphocyte-activation gene 3
MVI	macrovascular invasion
MASLD	metabolic dysfunction associated steatotic liver disease
MDSC	myeloid-derived suppressor cells
NK cell	natural killer cell
NR	not reached
ODAC	Oncologic Drugs Advisory Committee
ORR	overall response rate
OS	overall survival
PVR, CD155	poliovirus receptor
PVTT	portal vein tumour thrombosis
PD-L1	programmed cell death ligand 1
PD-L2	programmed cell death ligand 2
PD-1	programmed cell death protein-1
PFS	progression-free survival
Treg	regulatory T cell
TIM-3	T cell immunoglobulin domain and mucin domain 3
TIGIT	T-cell immunoreceptor with Ig and ITIM domains
FDA	The Food and Drug Administration
Vp4	thrombus in the main trunk of the portal vein or a contralateral portal vein invasion
TACE	transarterial chemoembolisation
TARE	transarterial radioembolisation
TRAE	treatment-related adverse event
TMB-H	tumour mutation burden-high
TAM	tumour-associated macrophage
TKI	tyrosine kinase inhibitor
AFP	α-fetoprotein
